# Development of Novel Micellar-Enhanced High-Throughput Microwell Spectrofluorimetric Method for Quantification of Lorlatinib: Application to In Vitro Drug Release and Analysis of Urine Samples

**DOI:** 10.3390/ph16091260

**Published:** 2023-09-06

**Authors:** Abdullah M. Al-Hossaini, Hany W. Darwish, Ahmed H. Bakheit, Ibrahim A. Darwish

**Affiliations:** Department of Pharmaceutical Chemistry, College of Pharmacy, King Saud University, P.O. Box 2457, Riyadh 11451, Saudi Arabia

**Keywords:** Lorlatinib, microwell-based methods, spectrofluorimetry, in vitro drug release, urine analysis

## Abstract

Lorlatinib (LOR) is a third-generation anaplastic lymphoma kinase (ALK) tyrosine kinase inhibitor drug. The Food and Drug Administration (FDA) has granted an approval for the use of LOR as a first therapeutic intervention for individuals diagnosed with ALK-positive metastatic and advanced non-small-cell lung cancer (NSCLC). The present study outlines, for the first time, the development and validation of an innovative microwell-based spectrofluorimetric (MW-SFL) method for the quantification of LOR. The proposed method involved the enhancement of the weak native fluorescence of LOR by its micellization into the sodium lauryl sulfate (SLS) micelles. The procedures of the method were conducted in white opaque plates with 96 microwells, and the enhanced fluorescence signals were measured by a fluorescence plate reader at 405 nm after excitation at 310 nm. The measured relative fluorescence intensity (RFI) had a linear relationship with LOR concentrations in the range of 60–1600 ng mL^−1^. The limit of detection (LOD) and the limit of quantification (LOQ) were found to be 19 and 56 ng mL^−1^, respectively. The method’s accuracy and precision were assessed using a recovery study; the recovery values ranged from 99.98% to 101.40%, accompanied by relative standard deviation (RSD) values of 0.42% to 1.59%. The proposed MW-SFL method combined the advantages of the intrinsically high sensitivity of the spectrofluorimetric measurement and the excellent throughput of the microwell-based approach. The results proved the method is effective in the determination of LOR in its pharmaceutical tablets, tablet dissolution testing, as well as in spiked urine with a high degree of precision and accuracy. The MW-SFL method is notable for its simple procedures and utilization of water as a solvent, as well as minimal quantities of sample solutions. These features align with its ecofriendly approach to green chemistry principles. These advantages gave the proposed MW-SFL method a high potential value for the determination of LOR in clinical and quality control laboratories.

## 1. Introduction

Cancer is the leading cause of mortality among men and women globally, as it poses a significant threat to global health. In 2020, there were an anticipated 18.1 million cancer cases worldwide. Lung cancer is the second type among all cancers in terms of prevalence. It is the most prevalent cancer among males and the second most prevalent among women. In 2020, there were more than 2 million new cases of lung cancer [1]. Small-cell lung cancer and non-small-cell lung cancer (NSCLC) are types of lung cancer. NSCLC accounts for ~85% of all lung cancers. The primary options for treating NSCLC include surgery, radiation, and chemotherapy. Generally, surgery and radiotherapy are beneficial for localized stage I and stage II NSCLC; however, they are not recommended for advanced NSCLC [2,3,4,5]. Chemotherapy is typically suggested as a superior first-line treatment for 80% of patients with NSCLC since it enhances life expectancy and quality of life [4,5,6]. Gefinitinib, erlotinib, and other first-generation tyrosine kinase inhibitors are frequently recommended [7,8]; however, a large proportion of patients have a chromosomal rearrangement that results in a fusion gene between anaplastic lymphoma kinase (ALK) and echinoderm microtubule-associated protein like 4 (EML4). The newly formed gene leads to the kinase protein’s constitutive activity, which subsequently enhances cellular malignancy [9,10,11]. The kinase activity of the fusion protein cannot be inhibited by medications from the first generation of tyrosine kinase inhibitors. As a result, the research in drug discovery fields was directed extensively to find new medications for the treatment of ALK-positive patients with NSCLC who are not responding to the earlier generations of ALK inhibitors.

**Figure 1:**
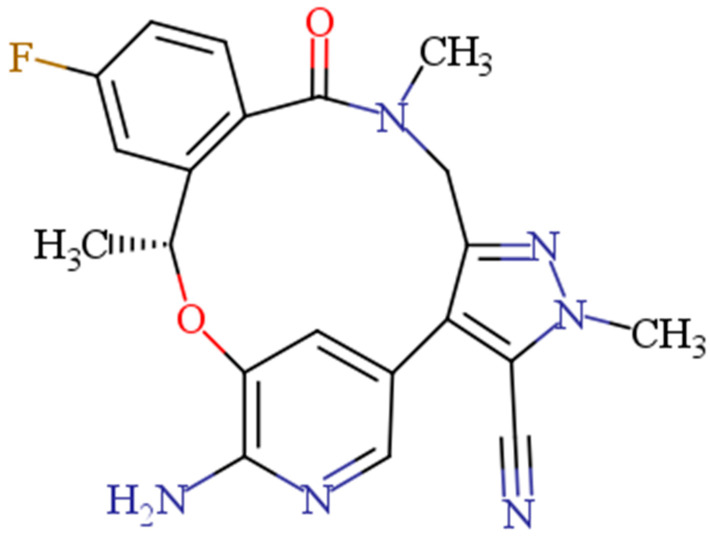
Chemical structure of lorlatinib (LOR).

Lorlatinib (LOR, Figure 1) is the first ALK inhibitor of the third generation [12,13]. Due to the clinical effectiveness of LOR, the US-FDA awarded regular authorization to LOR for patients suffering from advanced metastatic ALK-positive NSCLC, in 2021 [14]. Additionally, the European Medicines Agency (EMA) has also authorized LOR for the same indications [15]. Tablets containing LOR are sold commercially under the brand name Lorbrena^®^ (Pfizer Inc., New York, NY, USA). The suggested oral dose of LOR is 100 mg once a day [16].

The quality and safety of LOR’s therapeutic advantages are mainly dependent on the effectiveness of its pharmaceutical dosage forms (Lorbrena^®^ tablets). When the patent on Pfizer’s Lorbrena^®^ tablets expires, it is anticipated that the therapeutic success of LOR will encourage other pharmaceutical corporations to develop new pharmaceutical formulations for LOR. For the purposes of quality control, it would be necessary to determine LOR in its tablets. A suitable analytical method with high throughput is necessary to accomplish this goal. A survey of the literature revealed that liquid chromatography is the main analytical method available for determining LOR [17,18,19,20,21,22]. The majority of the currently available chromatographic techniques [18,19,20,21,22] rely on expensive tandem mass spectrometric detectors and are mainly validated for the analysis of biological specimens. The availability of simpler analytical methods that can be applied to the quantification of LOR in its bulk powder, dosage form (tablets), and biological specimens will be convenient. Our laboratory described two simple spectrophotometric methods for LOR via its charge transfer reactions [23,24]. These methods involved the charge transfer reactions of LOR with chloranilic acid and DDQ electron acceptors, and the employment of the reactions in developing spectrophotometric methods for LOR. These methods offered simple procedures; however, they are applicable only to the analysis of bulk powder and dosage form but not applicable to the analysis of biological fluids. The inapplicability of the previous spectrophotometric methods to the analysis of biological fluids was attributed to two main reasons. The first one is the interferences from water, being the electron donor, with the charge transfer reaction involved in the previous methods. The second reason was the low sensitivity of the previous methods that could not assess the low LOR concentrations in biological fluids. This limitation was due to their limited sensitivity and also the high interferences of aqueous biological samples because water contributes to the charge transfer reaction with LOR. For these reasons, it was very necessary to develop a highly sensitive method with a simple procedure and high throughput that is applicable to the quantification of LOR in bulk powder, dosage forms, and biological samples without interferences from either the pharmaceutical excipients or the aqueous matrix of the biological samples. This method is crucial for assessing the uniformity of pharmaceutical formulations, other pharmaceutical industry processes, and therapeutic drug monitoring [23,24,25].

This manuscript describes, for the first time, the development and validation of a microwell-based spectrofluorimetric method (MW-SFL) with high throughput for the determination of LOR in pharmaceutical quality control laboratories. The procedure of the proposed MW-SFL method is carried out in 96-microwell plates and involves measuring the fluorescence of the micelle between LOR and SLS in aqueous media by a fluorescence plate reader. The optimum experimental conditions of the method were optimized by changing one factor at a time strategy and, consequently, were successfully adopted for Lorbrena^®^ tablet analysis and extended for an in vitro drug release study, as well as LOR-spiked urine analysis. By virtue of aqueous medium domination in our study, the greenness of the proposed MW-SFL method is considered remarkable and offers extra advantage besides its high throughput.

## 2. Results and Discussion

### 2.1. Strategy for Method Development

**Figure 2:**
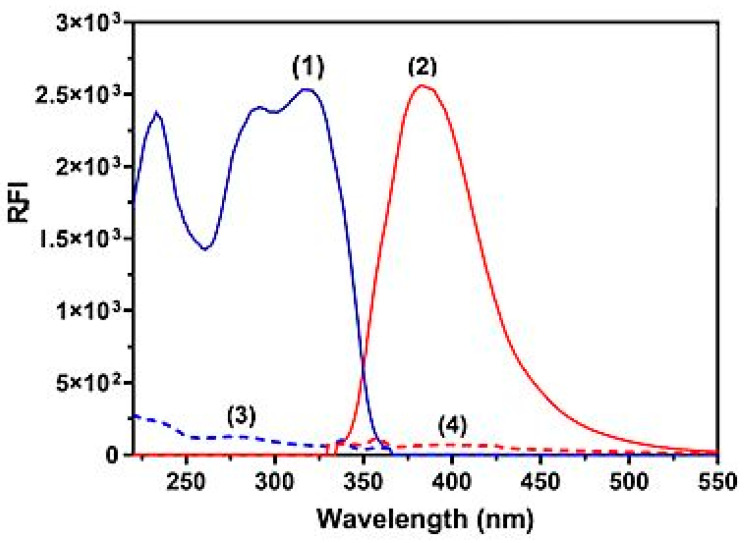
Excitation (spectra 1 and 3) and emission (spectra 2 and 4) spectra of LOR solutions (400 ng mL^−1^) in water (spectra 3 and 4) and in SLS medium (1%, *w*/*v*) (spectra 1 and 2).

In this study, LOR was chosen because of its therapeutic benefits to patients and a serious need for a reliable, accurate, and simple spectrofluorimetric method for measuring its amount in bulk powder and Lorbrena^®^ tablets. Figure 2 shows the LOR fluorescence spectra with and without SLS. Its excitation and emission peaks were at 310 and 405 nm, respectively. It was found that LOR’s native fluorescence was not strong enough to be used directly to construct a sensitive spectrofluorimetric method for measuring its concentration, especially in biological fluids such as urine. Thus, a micellar-enhanced method was used with SLS as a surfactant to increase the sensitivity so that it could be used to measure LOR in different matrices, such as dosage forms and biological fluids. Micellar-enhanced spectrofluorimetry had two main benefits: first, it could achieve high sensitivity, and second, it was a green and ecofriendly approach because the method took place in an aqueous medium. Traditional spectrofluorimetric methods have a low throughput [26] because they frequently have to be performed manually. Also, the harmful effects of the toxic organic solvents used in traditional analytical methods are passed on to the analyst, which is a more serious issue [27]. Several published reports have described the development of microwell-based spectrofluorimetric methods to measure the amount of different anticancer drugs in different sample matrices. These proposed methodologies have been utilized in fluorescence plate readers [28,29,30]. These ecofriendly methods exhibited high throughput, low sample volume, and low organic solvent use.

### 2.2. Optimization of Experimental Conditions

The present study investigated the impact of various conditions on the measured response, specifically the relative fluorescence intensity (RFI). Each condition was systematically altered while holding all other variables constant. The main variables subjected to investigations were found to be pH value, solvent and surfactant type, and volume for the sample. The RFI values were recorded at 405 nm after excitation at 310 nm.

#### 2.2.1. The Impact of Type and Volume of Surfactant

**Figure 3:**
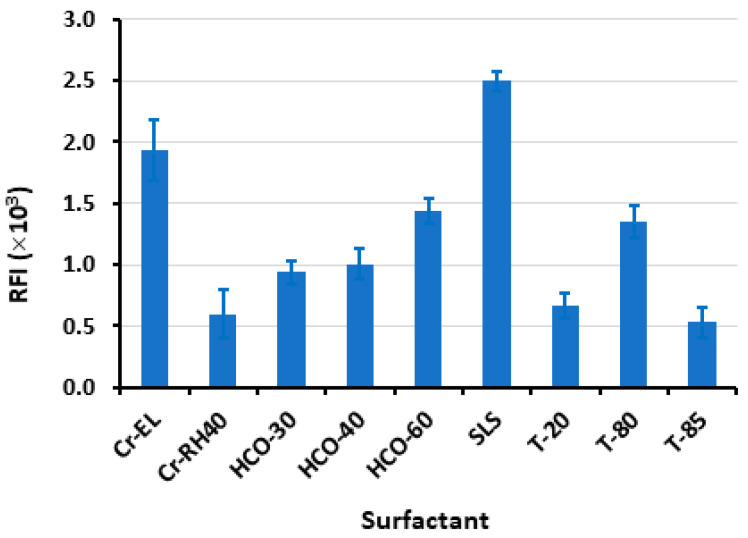
The effects of types of surfactant (1%, *w*/*v*) on the RFI of LOR (400 ng mL^−1^). Values are mean of 3 determinations ± SD.

**Figure 4:**
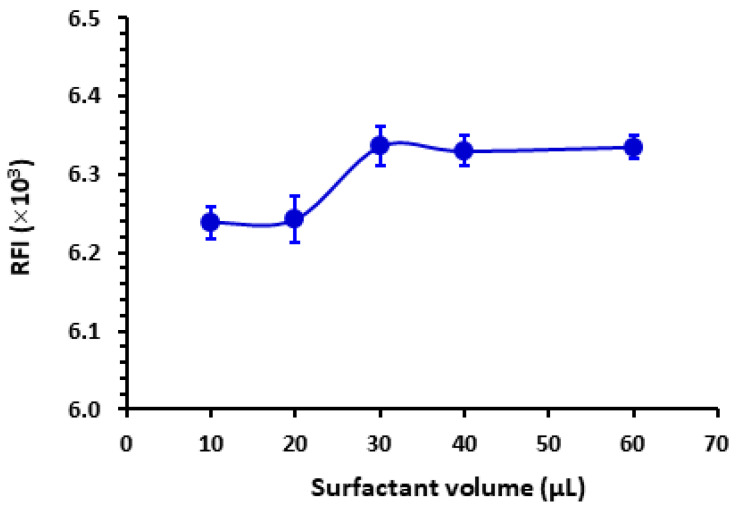
The effect of the volume of SLS (1%, *w*/*v*) on the RFI of LOR (1000 ng mL^−1^). Values are mean of 3 determinations ± SD.

The results depicted in Figure 3 indicate that the SLS yielded the highest RFI. Various volumes (ranging from 10 to 60 µL) of an aqueous solution containing 1% *w*/*v* of SLS were introduced into the LOR standard solution. SLS is indeed an anionic surfactant carrying a negative charge when dissolved in water due to the sulfate group present in its structure. This anionic nature of SLS contributes to its surfactant properties, such as its ability to lower surface tension and form micelles in aqueous solutions. According to Figure 4, the maximal response was attained utilizing 30 µL of SLS. Beyond this volume, no further increase in RFI was observed. For reading with better precision, a volume of 40 µL was utilized subsequently in this study. It is wise to mention that this experiment was conducted using a relatively high LOR concentration (4000 ng mL^−1^) that permitted the detection of the weak signals obtained with some surfactants such as T-85.

#### 2.2.2. The Impact of pH

**Figure 5:**
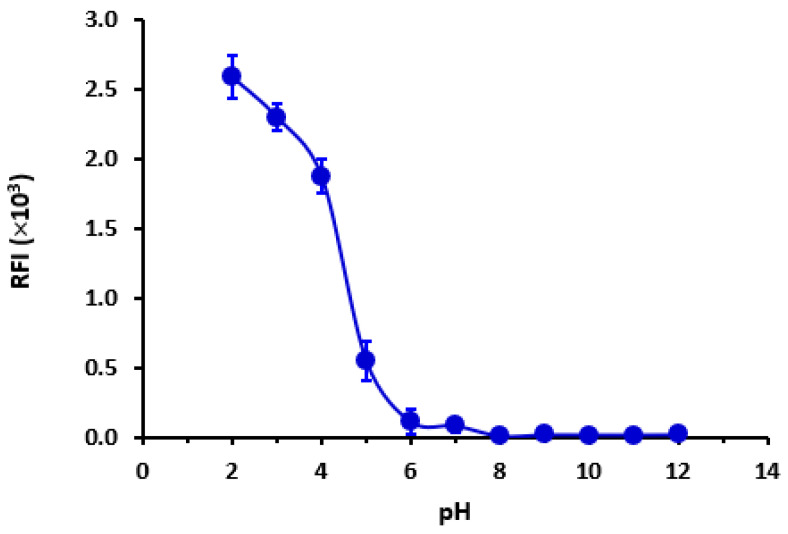
The impact of buffer pH on the RFI of LOR (400 ng mL^−1^) in SLS (1%, *w*/*v*) medium. Values are mean of 3 determinations ± SD.

**Figure 6:**
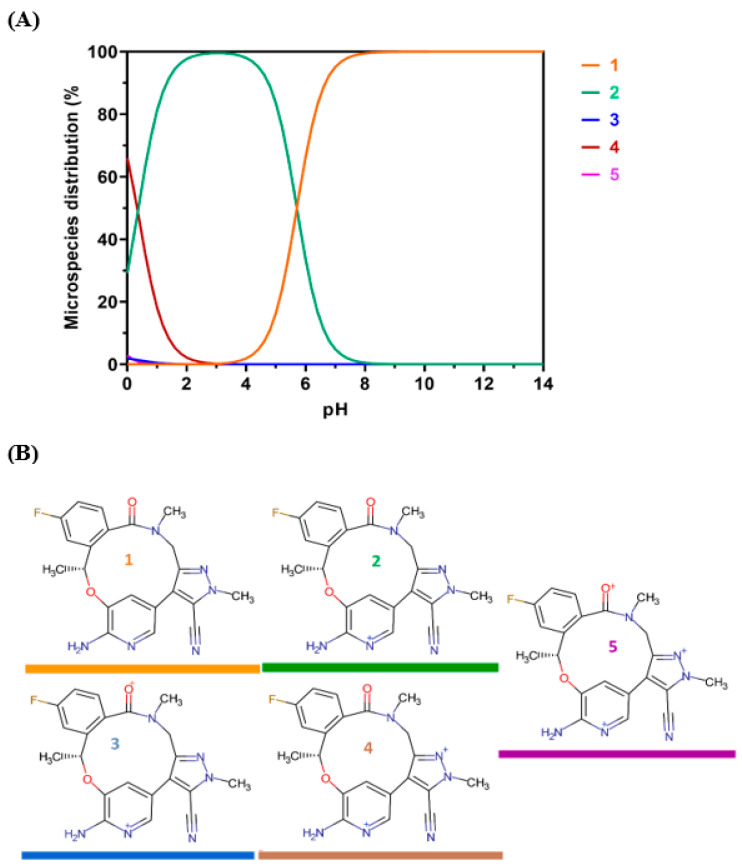
Theoretical protonation states of LOR as a function of the pH. (**A**): Microspecies distribution of LOR according to the pH value. The colors of the curves match with those of the chemical structures. (**B**): the chemical structures of the different protonated states of LOR.

The data indicate that the highest recorded RFI was observed at a pH of 2. As depicted in Figure 5, there was a significant decrease in RFI as the pH increased, as expected. Based on the available data, it was hypothesized that LOR assumes a cationic state at low pH levels, thereby facilitating favorable interaction with the negatively charged sulfonyl (OSO_3_^−^) group of SLS. The notion that the cationic form of LOR is the predominant form was corroborated by Chemicalize’s [31] computations that show the microspecies distribution of LOR versus pH (Figure 6). It was clear that at pH 2, LOR attained a positive charge, which confirms the previous hypothesis.

It was found that LOR molecules can exist in five different protonated states (Figure 6A). The major state of LOR is the cationic form, which exists at pH 2 (Figure 6B). This explains the strong interaction between cationic LOR and anionic SLS at pH 2.

#### 2.2.3. Effect of Diluent

**Figure 7:**
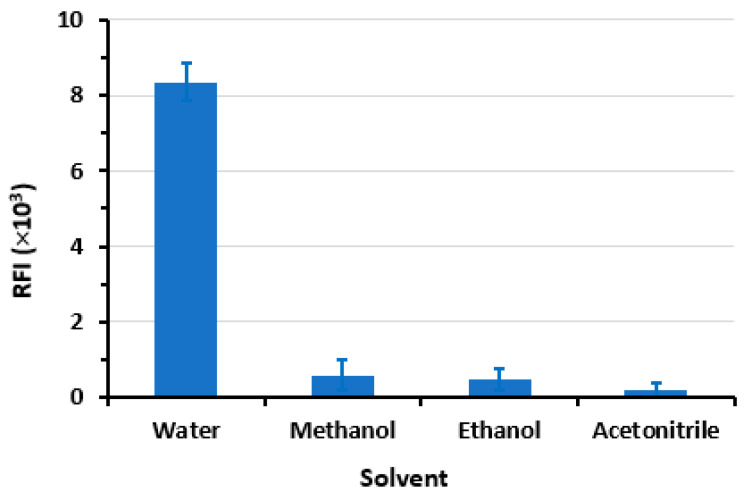
The impact of diluting solvent on the RFI values of LOR (1200 ng mL^−1^) in SLS (1%, *w*/*v*) medium. Values are mean of 3 determinations ± SD.

The findings of the study (Figure 7) indicate that water is the optimal solvent for dilution when SLS is present, as it yields the highest RFI and the lowest blank reading. Conversely, a noticeable and abrupt reduction in the RFI was observed in the micellar system when alternative solvents were employed. The observed phenomenon may be attributed to the denaturing impact of methanol and ethanol on the micelle that has formed. This can be attributed to the solubilization of these alcohols in water, which leads to a modification of the solvent properties, ultimately resulting in a reduction in the formation of micelles. Furthermore, the inclusion of these alcohols leads to a decrease in the dimensions of the micelles, yet with a gradual disintegration of the surfactant aggregate at exceedingly high concentrations [32,33,34].

#### 2.2.4. Effect of Time

To investigate the impact of time on the stability of the RFI of LOR in a micellar system, the RFI was observed over a period of 60 min. The results indicated that the RFI exhibited immediate development and maintained stable throughout the entire duration of the observation period (mean RFI values were 8.23 × 10^3^ ± 12.45).

### 2.3. Method Validation

#### 2.3.1. Linearity and Sensitivity

**Figure 8:**
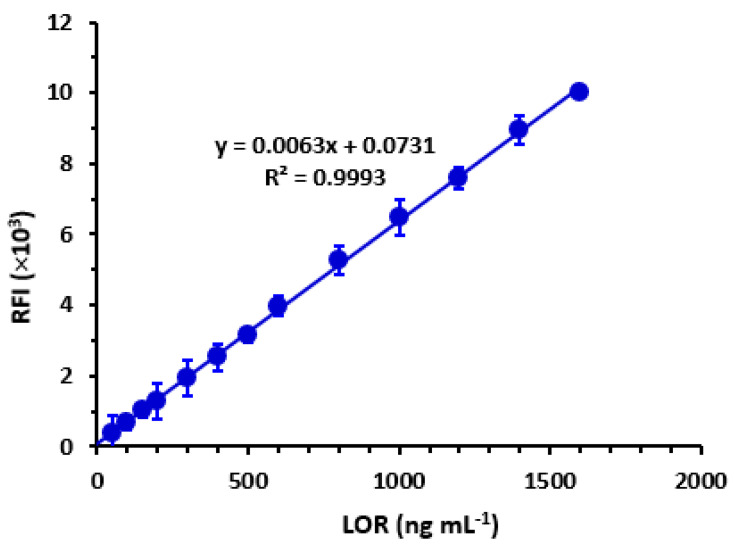
Calibration curve for the determination of LOR by the proposed MW-SFL method. Linear regression equation is given on the graph. Values are mean of 5 determinations ± SD.

The generation of a calibration graph involved plotting RFI values on the y-axis against corresponding LOR concentrations on the x-axis, measured in ng mL^−1^. The resulting data were subjected to regression analysis [35] using a weighting factor of 1. The results indicate that there exists a strong linear correlation (R^2^ = 0.9993) between the RFI values and LOR concentrations within the range of 60–1600 ng mL^−1^, as depicted in Figure 8. Table 1 presents the parameters utilized in the regression analysis. The determination of the limit of detection (LOD) and quantification (LOQ) was performed in accordance with the ICH Q2 (R1) guidelines [36]. LOD is defined as the lowest LOR concentration that can be detected by the proposed method. LOQ is defined as the lowest LOR concentration that can be quantified with acceptable accuracy and precision. The calculation of these parameters was carried out using the following formula:

$$LOD=3.3\;\frac{Sa}{b}\;and\;LOQ=10\;\;\frac{Sa}{b}$$

where *Sa* and *b* represent the standard deviation of the intercept of the regression line and the slope, respectively. Table 2 displays the nominated values for the limit of detection (LOD) and limit of quantification (LOQ) as 19 and 56 ng mL^−1^, respectively.

#### 2.3.2. Accuracy and Precision

The accuracy of the proposed methodology for the determination of LOR was evaluated by analyzing multiple samples of LOR bulk powder with three varying concentrations (low, medium, and high). These concentrations were 150, 800, and 1500 ng mL^−1^ on the same day (intraday accuracy) and over a period of three days in a row (interday accuracy). The recovery was calculated as the measured concentration to that of the taken, expressed as percentages. The findings presented in Table 2 indicate that the recovery percentages for intraday accuracy ranged from 99.98% to 101.34%, whereas the interday accuracy recovery percentages ranged from 99.02% to 102.46%. The recovery values, which were observed to be approximately 100%, serve as confirmation of the accuracy of the methodology employed for the quantification of LOR in its bulk form.

The precision of the method was evaluated by analyzing replicate samples of LOR at varying concentrations (150–1500 ng mL^−1^) in triplicate, both within and between assays. The RSD was used as a measure, calculated as the standard deviation relative to the mean, expressed as percentages. Table 2 displays the RSD values for intra- and inter-assay precisions, which were found to be 0.42–1.56% and 1.33–1.42%, respectively. The precision of the proposed methodology was deemed satisfactory based on the low RSD values obtained.

It is wise to mention that the obtained RSD and recovery values were within the acceptable limits of ICH for RSD (not more than 2%) and recovery percentages (97–103%) [36].

#### 2.3.3. Robustness

Table 3 presents the results obtained from deliberately altering several experimental variables. Based on the data, it is evident that the observed variations in the testing variables do not significantly affect the method’s accuracy and precision. This is supported by the recovery values, which ranged from 98.19 to 101.86% (±0.85–2.04). The proposed methodology is deemed robust based on the excellent results obtained, which indicate a mean recovery of approximately 100% and low RSD values.

#### 2.3.4. Specificity

The present study involved the observation of potential interferences arising from additives present in the LOR dosage form (Lorbrena^®^ tablets). The results indicated that there were no significant interferences, thereby highlighting the specificity of the adopted methodology, as presented in Table 4. The high specificity of the method was attributed to its measuring the specific fluorescence signal of LOR, whereas the common excipients that are used for tablets formulation are not fluorescent.

### 2.4. Applications of the Proposed MW-SFL Method

#### 2.4.1. Analysis of Lorbrena^®^ Tablets and Content Uniformity Testing

The proposed spectrofluorimetric method was employed to analyze Lorbrena^®^ tablets. The results indicate that the mean label percentage value was determined to be 99.14 ± 0.29%, as presented in Table 4. These results indicate the excellent accuracy of the proposed method and its suitability to be used in the quality control of pharmaceuticals, particularly in content uniformity testing.

#### 2.4.2. In Vitro Drug Release of Lorbrena^®^ Tablets

**Figure 9:**
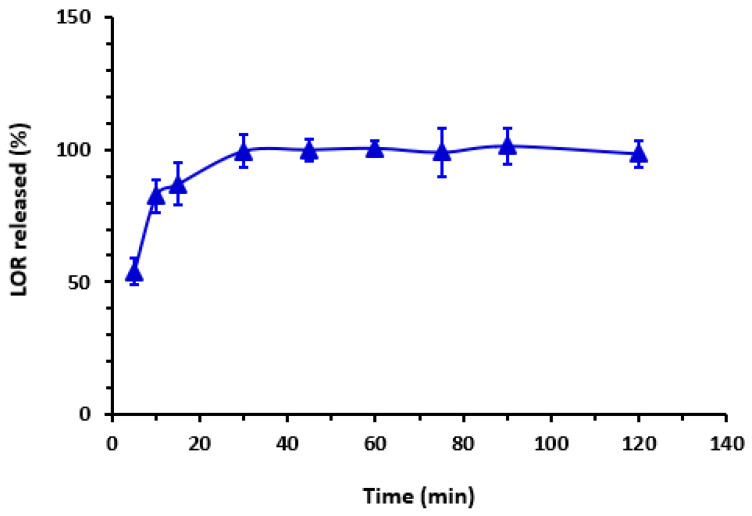
Impact of time on the dissolution of LOR tablets. Values are mean of 3 determinations ± SD.

A dissolution test, also known as an in vitro drug release test, was conducted on 100 mg tablets (three tablets) of Lorbrena^®^. The concentration of the released LOR was determined using the regression linear equation and the RFI of the cited drug at 405 nm. The dissolution time was utilized to monitor the percentage of drug released, as indicated in Figure 9. The study revealed that about 100% of LOR was released within a 30 min timeframe, a result that aligns with the acceptable standards outlined in the USP guidelines (refer to Figure 9).

#### 2.4.3. Analysis of Urine Samples

In order to evaluate the attainable feasibility of the present methodology for the analysis of urine samples comprising LOR, an estimation was made regarding the quantity of LOR that is excreted in an unaltered state in the urine, which was found to be approximately 1 mg/day [15]. Consequently, the expected LOR concentrations were deliberately introduced into urine samples, subsequently subjected to processing, and, ultimately, diluted to attain the specified LOR concentrations falling within the linear range of the methodology, namely, 50–1600 ng mL^−1^. Following the examination of these specimens, the average recovery percentage for LOR was determined to be 100.15 ± 3.32%, as presented in Table 4. According to the standards for bioanalytical technique validation, the suggested MW-SFL method for LOR assessment in urine samples has an excellent level of accuracy [36].

### 2.5. Greenness Assessment

Microwell plate assays assisted with microplate readers are generally considered to adhere to the principles of green analytical chemistry (GAC) due to their low solvent and reagent consumption and minimal waste production. To evaluate the greenness of the proposed MW-SFL method for LOR, two recent metric tools were employed: green analytical procedure index (GAPI) [37] and analytical greenness (AGREE) [38].

**Figure 10:**
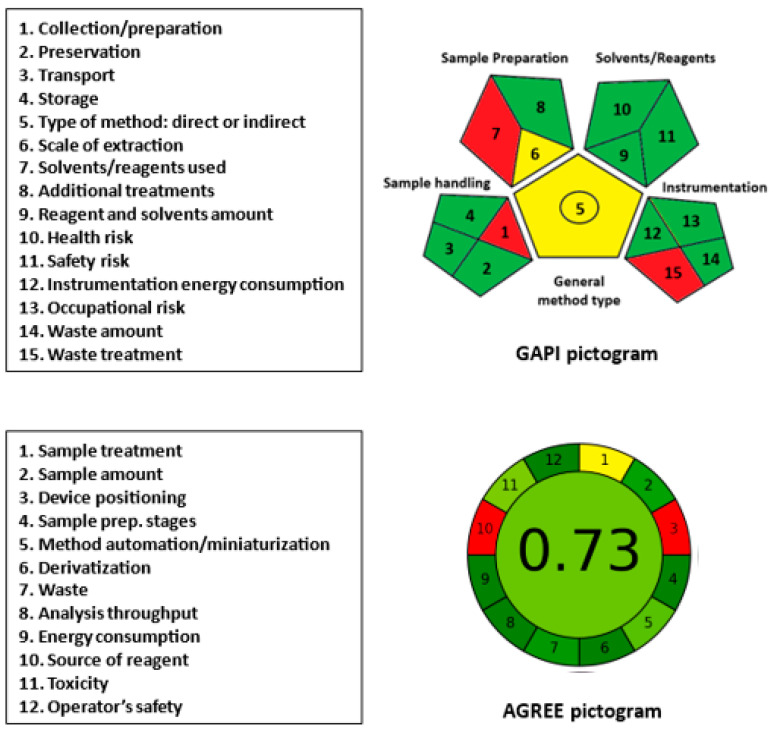
Results of GAPI and AGREE analysis for evaluation of the greenness of the proposed MW-SFL method for the determination of LOR.

The GAPI tool [37] provides a comprehensive assessment of the ecological influence of the analytical procedures. It evaluates the method greenness via 15 parameters classified into 5 categories, including sample handling, sample preparation, solvent/reagent usage, instrumentation, and assay type. The results are presented as a pictogram consisting of 15 sections, each assigned a color code (green, yellow, or red), with green representing a safe procedure and red indicating a non-green procedure. In the GAPI pictogram (Figure 10), three parameters (1, 7, and 15) related to sample collection/preparation, the use of solvents/reagents, and waste treatment were red. The use of an off-line sample collection/preparation, methanol and SLS, and the absence of treatment for method waste contributed to these results.

The AGREE tool [38] is a novel approach to evaluate the greenness of analytical procedures. This user-friendly software evaluates 12 parameters based on the principles of GAC and provides comprehensive, flexible, informative, and easily interpretable results. The resulting analysis is automatically generated and presented as a circular pictogram comprising 12 sections, each assigned a specific color code indicating the score, ranging from deep green (score = 1) to deep red (score = 0). The overall score, which is a fraction of unity, is automatically calculated and displayed at the center of the pictogram. Overall, the AGREE metric tool is a valuable and efficient method for evaluating the eco-friendliness of analytical procedures. In the AGREE pictogram (Figure 10), parameter 3 (device positioning) was assigned as red due to off-line sample treatment and the non-automated operation of the micro-plate reader. Parameter 10 (source of reagent, if any) was assigned red because none of the reagents were from bio-based sources. The remaining parameters were marked as yellow or varying degrees of green. Parameter 11 (volume of reagent/solvent) and parameter 12 (operator’s safety) were assigned green because only 0.2 mL of sample and reagents was used per sample (parameter 11), and the entire procedure was safe to the operator (parameter 12). The overall assessment score, automatically generated in the center of the AGREE pictogram, was 0.73 out of 1, indicating the overall acceptable greenness of the proposed assay.

In conclusion, the proposed MW-SFL method conforms to the requirements of a green analytical approach to routine use for the analysis of LOR in both dosage forms and urine samples. The greenness evaluation of the method, as demonstrated by the GAPI and AGREE pictograms, supports its environmental sustainability and suitability for routine use in pharmaceutical and clinical analysis.

## 3. Materials and methods

### 3.1. Apparatus

Recording the fluorimetric spectra was conducted by a fluorescence spectrometer (model FP-8200; manufacturer: Jasco Corporation, Tokyo, Japan). A slit width of 5.0 nm was employed for both the excitation and emission monochromators. The instrument was routinely calibrated to ensure that it maintained its linearity by using the standard quinine sulfate at a concentration of 0.01 g mL^−1^. Additionally, in order to calibrate it in terms of wavelength, λ_ex_ was measured at 292 nm, and λ_em_ was measured at 420 nm (as instructed by the instrument manufacturer). The equipment was supplemented with SpectraManager^®^ software, which was responsible for the fluorescence spectra being translated into an ASCII format. Microplate fluorescence reader (FLx800: Bio-Tek Instruments Inc., Winooski, VT, USA) assisted by KC junior software, which was supplied along with the device, was used for measuring the fluorescence signals in microwell plates at 405 nm after excitation at 310 nm. For the purpose of adjusting the pH of the solutions, a pH meter (Hanna: Romania) was utilized. For the purpose of dissolution testing, an automatic dissolution apparatus with an 8-cup system was purchased from Abbott Corporation in Princeton, NJ, USA.

### 3.2. Reagents and Materials

Lorlatinib (LOR) of purity 99.73% (assigned by the supplier) was utilized throughout the study (Selleck Chemicals, TX, USA). The Saudi Food and Drug Authority in Riyadh (SFDA), Saudi Arabia, generously offered Lorbrena^®^ tablets manufactured by Pfizer Inc. in New York, USA. Each tablet was labeled to indicate that it contained 100 mg of LOR. Sodium lauryl sulfate (SLS; 95%) was purchased from Winlab (Pontefract, London, UK) and prepared as 1% (*w*/*v*) aqueous solution. Other surfactants such as Cremophor RH 40 and Cremophor EL were procured from BASF (Ludwigshafen, Germany) and prepared as 1% (*v*/*v*) aqueous solutions. Components of the buffer were of an analytical grade, including various acids, such as phosphoric, citric, and boric acids, in addition to other components, including KH_2_PO_4_, Na_2_HPO_4_, KCl, and NaOH. Sigma-Aldrich (St. Louis, CA, USA) was the vendor for the acquisition of solvents such as methanol, ethanol, and acetonitrile. A Milli-Q plus water purification system was utilized in obtaining the purified water (Merck KGaA, Darmstadt, Germany). Every day, freshly made buffer solutions were created, with pH levels ranging from 2 to 12.

Merck & Co., Inc., was the vendor for the procurement of Corning^®^ 96–well white opaque polystyrene assay plates with flat bottoms (Rahway, NJ, USA). Thermo Fisher Scientific Inc. supplied us with Finnpipette^TM^ pipettes that were both adjustable on a single channel and on eight channels (Waltham, MA, USA). Merck KGaA (Darmstadt, Germany) was the vendor for the acquisition of reagent reservoirs (BRAND^®^ PP) with cover lids for the purpose of dispensing solutions using 8-channel pipettes.

### 3.3. Preparation of Standard and Sample Solutions

#### 3.3.1. Standard Solutions

In methanol, an LOR stock solution (1 mg mL^−1^) was prepared and then diluted with methanol to prepare a working LOR solution (1 μg mL^−1^). Both stock and working solutions were determined to be at least stable for 14 days when kept in a refrigerator (8 °C).

#### 3.3.2. Tablet Sample Solution

Ten Lorbrena^®^ tablets were crushed into a fine powder and then weighed. Powdered tablet equivalent to 100 mg of LOR was weighed and then transferred to a 50 mL calibrated flask. After adding about 20 mL of methanol, the contents were sonicated for 10 min and then shaken for 15 min to ensure complete dissolution. After this, methanol was added to a mark (50 mL). After filtering the solution that was produced, the initial part of the filtrate that was collected was thrown away. Following diluting the filtrate with methanol, the LOR concentrations were brought down to a range that went from 60 to 1600 ng mL^−1^. These solutions were analyzed for their nominated LOR concentrations using the MW-SFL method.

#### 3.3.3. Human Urine Samples

Human drug-free urine (1 mL) was spiked with a volume of 20 µL of standard LOR solutions (of different concentrations) and mixed for 60 s, 1 mL of methanol was added, and the mixture was vortexed for 30 s; the samples were then centrifuged at 12,000 rpm for 15 min at room temperature for protein precipitation and separation. These conditions were recommended by the centrifuge manufacturer’s instructions.

The supernatants were transferred into glass vials and dried under a gentle stream of nitrogen. Reconstitution of the residue was conducted by adding methanol, then suitable dilutions were made to give a final concentration range of 60–1600 ng mL^−1^. The obtained diluted samples were subjected to analysis by the proposed method. A blank urine sample was treated similarly. Fluorescence intensity was measured at 405 nm after excitation at 310 nm and LOR concentration was determined from corresponding regression equation in urine media.

### 3.4. Optimization of Conditions

The impact of surfactant type on the relative fluorescence intensity (RFI) values of LOR was investigated by introducing 40 µL of an aqueous solution (1%, *w*/*v*) of each specific surfactant to the wells containing 100 µL of the LOR solution (400 ng mL^−1^). The surfactants employed in the study were Cremophor RH 40 (Cr RH-40); Cremophor EL (Cr-EL); polyoxyethylene hydrogenated castor oil 30, 40, and 60 (HCO -30, 40, and 60); Tween 20 (T-20), T-80, and T-85; and SLS.

The impact of pH was investigated through the introduction of various buffers into the LOR solution (400 ng mL^−1^). The entire pH range was encompassed through the utilization of 0.1 M acetate buffer and 0.1 M borate buffer separately.

The impact of various diluting solvents, namely, water, methanol, ethanol, or acetonitrile, on the RFI of LOR (1200 ng mL^−1^) in 1% *w*/*v* SLS was examined.

### 3.5. Recommended Procedures

Samples (standard solution, tablet solutions, and urine) containing LOR concentrations of 60, 100, 200, 250, 300, 400, 500, 600, 800, 1000, 1250, 1400, and 1600 ng mL^−1^ were prepared and used to construct the calibration experiments. Aliquots of LOR working standard solutions, 40 µL of acetate buffer solution (pH 2), and 40 µL of aqueous SLS (1% *w*/*v*) were added, and the volume was completed to 200 µL with water into the wells of 96-microwell assay plates. The response (RFI) was measured at 405 nm for emission after being excited at 310 nm. On the graph, the response values were displayed along the y-axis, while the LOR concentrations were plotted along the x-axis. An evaluation of the data using regression was carried out, and thereafter, a regression equation was constructed.

### 3.6. In Vitro Dissolution Testing for Lorbrena^®^ Tablets

In vitro dissolution of Lorbrena^®^ tablets was performed as directed by the FDA procedure of dissolution [39]. A volume of 900 mL of acetate buffer (pH 4.5) at 37.5 ± 0.5 °C was used for the dissolution of the Lorbrena^®^ tablets. Samples (5 mL) of Lorbrena^®^ tablet solution were taken and passed through 0.45 μm syringe filter. Sample volume (5 mL) was replaced by adding the same volume of the dissolution medium. The samples were diluted and analyzed, as described under “Recommended Procedures”.

## 4. Conclusions

A novel method was developed and evaluated to determine LOR in different matrices. This method involved the use of a microwell-based spectrofluorimetric technique, which was assisted by a fluorescence plate reader. The weak native fluorescence of LOR was enhanced by the addition of a surfactant to increase the method sensitivity for enabling the quantification of low LOR concentrations in urine samples. In contrast to prior methodologies, the current approach is distinguished by its expeditiousness, straightforwardness, and utilization of minimal sample quantities. The methodology additionally provided a substantial throughput, thereby enabling the expeditious and effortless processing of a multitude of samples in clinical or quality control laboratories. In addition, the technique is economically efficient as it is based on the assessment of LOR’s inherent fluorescence, rather than the utilization of costly derivatizing agents. The present methodology was efficacious in the analysis of pharmaceutical tablets and urine. The method has been customized for LOR tablet dissolution testing, rendering it a practical and uncomplicated approach. This approach is being praised as a positive move toward sustainability and cost-efficiency, as it is ecofriendly and may serve as a substitute for existing LOR analytical methods, which are liquid chromatography with limited throughputs and spectrophotometry with limited sensitivity.

## Figures and Tables

**Table 1:** Analytical data of the MW-SFL method for determination of LOR.

Parameter	Value
Wavelength; λ_ex/_λ_em_ (nm)	310/405
Linearity range (ng mL^−1^)	60–1600
Intercept (*a*)	73.10
Slope (*b*)	6.30
Correlation coefficient (*r*)	0.9994
Standard deviation of intercept (*S_a_*)	±35.40
Standard deviation of slope (*S_b_*)	±0.0453
Limit of detection (ng mL^−1^)	19
Limit of quantification (ng mL^−1^)	56

**Table 2:** Accuracy and precision data for the determination of LOR using the MW-SFL method.

Amount Taken (ng mL^−1^)	% Found	% RSD
Intraday		
150	99.98 ± 0.42	0.42
800	100.25 ± 1.56	1.56
1500	101.34 ± 1.24	1.24
Interday		
150	99.02 ± 1.32	1.33
800	101.62 ± 1.42	1.42
1500	102.46 ± 1.35	1.35

**Table 3:** Robustness of the proposed MW-SFL method for determination of LOR.

Practical Paramete	Recovery (% ± SD) ^a^
No variation ^b^	99.92 ± 0.85
pH variation	
1.8	99.81 ± 1.31
2.2	98.24 ± 2.04
Buffer volume (µL)	
38	98.19 ± 1.19
42	101.86 ± 1.44

^a^ Average of three determinations; ^b^ Calibration procedure was used.

**Table 4:** Application of proposed MW-SFL method for determination of LOR in bulk powder, urine samples, and Lorbrena^®^ tablets.

Bulk Powder			Tablets			Urine		
Taken Conc. (ng mL^−1^)	Found Conc. (ng mL^−1^)	Recovery (%)	Added Conc. (ng mL^−1^)	Found Conc. (ng mL^−1^)	Recovery (%)	Added Conc. (ng mL^−1^)	Found Conc. (ng mL^−1^)	Recovery (%)
60	59.95	99.92	60	59.36	98.94	60	61.62	102.70
100	99.47	99.47	100	99.47	99.47	75	72.30	96.40
200	199.73	99.87	200	198.01	99.00	100	101.35	101.35
	Mean	99.75	Mean		99.14		Mean	100.15
	±SD	0.25	±SD		0.29		±SD	3.32

## Data Availability

Data is contained within the article.
